# Current status and future prospects of nanocarrier-mediated miRNA delivery for osteoarthritis therapy

**DOI:** 10.3389/fmed.2025.1728944

**Published:** 2026-01-15

**Authors:** Longyin Li, Zhengguang Xu, Feng Gao, Junjie Xu

**Affiliations:** 1 Department of Joint Surgery, The Fourth Affiliated Hospital of Anhui Medical University, Chaohu, China; 2 School of Clinical Medicine, Jining Medical University, Jining, China

**Keywords:** gene therapy, microRNAs, nanoparticles, osteoarthritis, targeted delivery

## Abstract

Osteoarthritis (OA) is a common degenerative joint disease whose pathogenesis involves multiple pathways, including inflammatory responses, cartilage matrix metabolism, cell proliferation, and apoptosis. Currently, effective clinical treatments are lacking. MicroRNAs (miRNAs) are associated with the pathogenesis of OA and represent potential therapeutic agents for this disease. However, issues such as miRNA instability, off-target effects, and low cellular uptake efficiency have limited their clinical application. Nanocarriers, which are widely used for targeted drug delivery, offer a convenient approach for miRNA-based OA therapy. Numerous studies have employed nanomaterials such as polymer-based, lipid-based, inorganic nanoparticles, and extracellular vesicles (EVs) to deliver miRNAs, effectively inhibiting the progression of OA and achieving therapeutic goals. This review summarizes research advances in the use of nanoparticles to deliver miRNAs for the treatment of OA, explores the associated clinical prospects and challenges, and proposes potential pathways toward intelligent, precise, and personalized therapy, with the aim of informing miRNA-mediated gene therapy for OA.

## 1 Introduction

Osteoarthritis (OA) is a chronic, degenerative whole-joint disease that affects all joint tissues (1). Its pathological changes involve the cartilage, synovium, subchondral bone, menisci, ligaments, joint capsule, and periarticular adipose tissue, including the infrapatellar fat pad (2–4). OA causes significant pain, functional limitations, and a reduced quality of life (5). It ranks as the fourth leading cause of disability globally, affecting more than 500 million people worldwide. The prevalence of OA is projected to increase by 60–100% by 2050 (1, 3, 6). The major risk factors for OA include aging, female sex, obesity, a history of joint injury, abnormalities in joint alignment, and genetic as well as metabolic factors (7). Currently, OA treatment focuses primarily on alleviating pain symptoms, with no effective methods available to slow disease progression (8).

MicroRNAs (miRNAs) are a class of endogenous single-stranded RNAs approximately 22 nucleotides in length that play crucial roles in regulating cartilage homeostasis, inflammatory responses, and cell proliferation and apoptosis (9, 10). Delivering miRNAs for the treatment of OA has broad application prospects (11). However, owing to issues such as poor *in vivo* stability, weak tissue targeting, and low cellular uptake rates of miRNAs, direct delivery into the body diminishes therapeutic efficacy, severely limiting their application (12–14). Therefore, it is necessary to construct a drug delivery system that can protect miRNAs, ensure targeted delivery, enhance their efficacy, and accelerate their clinical application (15).

In recent years, biologics and targeted therapies have emerged as novel treatment strategies for OA, including novel drug delivery systems (16, 17). Using nanomaterials as carriers for miRNA delivery leverages the inherent advantages of nanocarriers while effectively circumventing the inherent limitations of the miRNAs themselves (18, 19). Currently, various types of nanocarriers are used for the delivery of miRNA (20). In this narrative review, we summarize recent findings on miRNA delivery via nanoparticles for treating OA, explore the similarities and differences among various types of carriers, and outline future development directions and prospects for clinical translation, thereby providing a reference for subsequent research.

## 2 miRNA biogenesis

**Figure 1:**
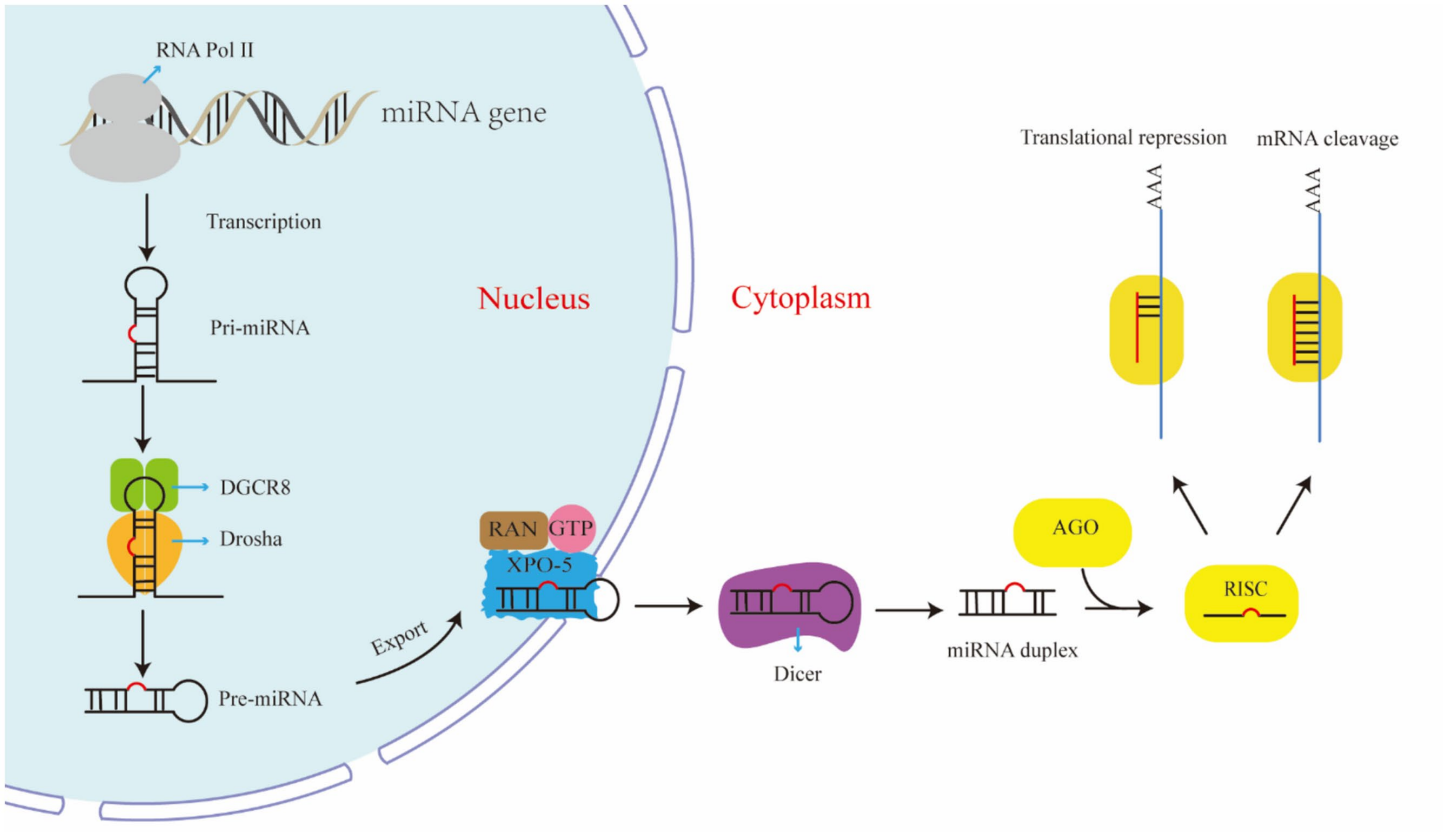
Canonical miRNA biogenesis. Briefly, RNA polymerase II transcribes miRNA genes to produce pri-miRNA, which is cleaved into pre-miRNA by the Drosha-DGCR8 complex in the cell nucleus. They are then exported from the nucleus to the cytoplasm via Exportin-5 (XPO5). In the cytoplasm, they are further cleaved by the Dicer enzyme into RNA duplexes, where the guide strand is incorporated into the RNA-induced silencing complex (RISC). The RISC guide complex targets mRNAs, leading to their degradation or translational repression.

The biogenesis of miRNAs is not only essential for normal cellular processes, but is also closely associated with the onset and progression of various diseases (21). Understanding the miRNA biogenesis pathway and its intricate regulatory network has profound implications for elucidating the role of miRNAs in the pathogenesis of OA and exploring potential therapeutic targets. The biogenesis of miRNAs is a complex and precisely regulated multistep process that is divided mainly into the canonical pathway and the noncanonical pathway. The canonical miRNA biosynthetic pathway primarily originates from transcription by RNA polymerase II within the cell nucleus. The resulting primary miRNA (pri-miRNA) can extend to several thousand nucleotides in length and typically contains one or more hairpin structures (10). Pri-miRNAs are recognized and cleaved by the Drosha-DGCR8 complex within the nucleus to produce precursor miRNAs (pre-miRNAs). The newly generated pre-miRNA is actively transported out of the nuclear pore by Exportin-5 (XPO5) in a Ran-GTP-dependent manner (22). Once inside the cytoplasm, the Dicer enzyme and its cofactor TRBP cleave the pre-miRNA into small miRNA duplexes. These duplexes are loaded onto Argonaute (Ago) proteins, where one strand is retained as the guide strand to form the RNA-induced silencing complex (RISC), while the other passenger strand is removed or degraded (23). When the RISC hybridizes with target mRNA, it induces mRNA degradation or translation inhibition (24) (Figure 1). While most miRNAs are processed in this way, a small number of miRNAs bypass certain steps in the canonical pathway to be processed in a noncanonical manner (25, 26).

## 3 Role of miRNAs in the development of osteoarthritis

In the normal joint microenvironment, the extracellular matrix (ECM) maintains a dynamic balance between synthesis and degradation, inflammation and anti-inflammation, apoptosis and proliferation, and various biological responses, collectively ensuring the homeostasis of joint structure and function (27–29). The pathological core of OA lies in the disrupted homeostasis of anabolic and catabolic signaling networks within the joint (30). Among the numerous molecular mechanisms involved in this imbalance, miRNA-mediated post-transcriptional regulation has gradually attracted increasing attention (Table 1).

**Table 1:** The role of the selected miRNAs in the development of osteoarthritis.

miRNA	Potential targets	*In vivo*/*in vitro* trial	Animal models	Cell models	Role in the progression of OA	References
miR-653-5p	IL-6	*In vivo* and *in vitro*	Mice	Primary human chondrocytes, C28/I2	↓ *	(33)
miR-149	VCAM-1	*In vivo* and *in vitro*	Mice	–	↓	(34)
miR-22-3p	NLRP3	*In vivo* and *in vitro*	Mice	C28/I2	↓	(35)
miR-4701-5p	HMGA1	*In vitro*	–	CHON-001	↓	(36)
miR-214-3p	IKKβ	*In vivo* and *in vitro*	Mice	Primary human and mouse chondrocytes	↓	(37)
miR-548d-5p	SP1	*In vivo* and *in vitro*	Rats	C28/I2	↓	(38)
miR-485-3p	Notch2	*In vitro*	–	SW1353 and CHON-001	↓	(39)
miR-140-5p	Jagged1/HMGB1	*In vivo* and *in vitro*	Rats	C28/I2, cartilage progenitor/stem cells and terminally differentiated cartilage chondrocyte	↓	(40, 41)
miR-5581	NRF1	*In vivo* and *in vitro*	Mice	Primary human chondrocytes	↑ *	(43)
miR-27b-3p	PPARG	*In vivo* and *in vitro*	Mice	Human OA fibroblast-like synoviocytes	↑	(44)
miR-128a	NR1D2	*In vivo* and *in vitro*	Mice	Primary mouse chondrocytes	↑	(45)
miR-33-5p	SIRT6	*In vivo* and *in vitro*	Rats	Primary human chondrocytes	↑	(42)
miR-18a-3p	PDP1/HOXA1	*In vivo* and *in vitro*	rats, mice	ATDC5, primary human and mouse chondrocytes	↑/↓	(46, 47)
miR-146a-5p	CXCR4/NUMB	*In vivo* and *in vitro*	Rabbits, rats	C28/I2, primary human and mouse chondrocytes	↑/↓	(48, 49)

^*^↑, intensification. ↓, delay.

### 3.1 miRNAs with protective roles in OA

Extensive research has indicated that certain miRNAs are significantly downregulated in the cartilage tissue of OA patients, thereby promoting disease progression. The overexpression of these miRNAs can protect against OA by regulating the biological functions of chondrocytes and immune cells (31, 32). For example, Lin et al. (33) fully confirmed through clinical samples and animal experiments that miR-653-5p inhibits the JAK/STAT3 signaling pathway by targeting and suppressing IL-6 expression. Inactivation of this pathway alleviates chondrocyte senescence by downregulating expression of the cyclin-dependent kinase inhibitors p21 and p16^INK4a^, while simultaneously suppressing matrix metalloproteinase-13 (MMP13) and a disintegrin and metalloproteinase with thrombospondin motifs 5 (ADAMTS5), thereby significantly mitigating the progression of OA pathology. Research has revealed that miR-149 directly targets and suppresses VCAM-1 expression, thereby inhibiting the activation of the PI3K/AKT signaling pathway. The inactivation of this pathway reduces inflammation by downregulating NF-κB activity, regulates the balance between Bax and Bcl-2 to inhibit chondrocyte apoptosis, and promotes the synthesis of ECM components such as collagen II and aggrecan, thereby collectively alleviating OA. Moreover, upregulating miR-149 or inhibiting VCAM-1 in *in vivo* models can significantly improve the pathological manifestations of OA, suggesting that the miR-149/VCAM-1/PI3K/AKT axis may represent a potential therapeutic pathway for OA treatment (34). Lu et al. (35) reported that the expression of miR-22-3p is downregulated in OA, promoting disease progression, and they demonstrated through *in vitro* and *in vivo* experiments that miR-22-3p alleviates OA progression by inhibiting the expression of the inflammasome NLRP3. Additionally, bioinformatics analysis and dual luciferase reporter assays confirmed that HMGA1 is a target of miRNA-4701-5p. By targeting and suppressing HMGA1 expression, miRNA-4701-5p alleviates IL-1β-induced inflammatory damage, apoptosis, and cytotoxicity in chondrocytes (36). Another study reported that miR-214-3p directly targets IKKβ to inhibit the NF-κB signaling pathway, thereby reducing the expression of MMP3, MMP13, and the apoptotic executor protein cleaved-caspase-3, while concurrently elevating the levels of the anti-apoptotic protein Bcl-2. This mechanism alleviates the degradation of the cartilage ECM and apoptosis; moreover, animal experiments have confirmed that intra-articular injection of miR-214-3p agonists can slow the progression of OA (37). Furthermore, miR-548d-5p, miR-485-3p, and miR-140-5p also promote chondrocyte proliferation and inhibit apoptosis (38–41).

### 3.2 miRNAs with pathogenic roles in OA

In the pathological environment of OA, multiple miRNAs amplify inflammatory responses, promote chondrocyte senescence and apoptosis, thereby disrupting joint homeostasis and driving OA progression. For example, Liu et al. (42) reported that miR-33-5p targets and suppresses SIRT6, thereby leading to the upregulation of cell cycle–dependent kinase inhibitors such as p16 and p21 and a weakening of the inhibitory control over the NF-κB signaling pathway. These changes induce chondrocyte senescence, promote inflammation, and trigger cell cycle arrest, ultimately leading to cartilage degeneration and OA progression. This effect was further validated in animal experiments. Similarly, miR-5581 is upregulated in the cartilage tissue of OA patients. Bioinformatics prediction and dual luciferase reporter assays confirmed that its targeting reduces nuclear respiratory factor 1 protein levels, thereby inhibiting cell proliferation, promoting apoptosis, and disrupting ECM homeostasis (43). Tavallaee et al. (44) reported that miR-27b-3p is upregulated in OA, accelerating OA progression by promoting synovial fibrosis via the PPARG/ADAMTS8 axis. In addition, miR-128a exacerbates OA progression by targeting and suppressing nuclear receptor subfamily 1 group D member 2 (NR1D2), leading to decreased expression of chondrogenic factors and reduced ECM synthesis, whereas cartilage-specific knockout of miR-128a can restore NR1D2 expression and significantly alleviate OA severity (45).

### 3.3 Dual-functioning miRNAs in the progression of osteoarthritis

Owing to the complex interaction networks between miRNAs and RNA, some miRNAs do not exert purely protective or inhibitory effects but instead exert differential influences on the progression of OA through distinct signaling pathways. For example, miR-18a-3p can target and inhibit PDP1, thereby reducing the expression of inflammatory cytokines and matrix metalloproteinases, improving cartilage matrix remodeling, and suppressing inflammatory responses (46). However, Ding et al. (47) reported that miR-18a-3p can suppress HOXA1 expression, leading to the upregulation of cleaved caspase-3 and cleaved PARP and the downregulation of BCL-2 expression. This promotes chondrocyte apoptosis and drives the pathological progression of OA. In addition, studies have shown that miR-146a-5p can simultaneously target and inhibit the expression of CXCR4 and NUMB, the former of which blocks SDF-1/CXCR4-mediated chondrocyte autophagy, thereby reducing cartilage degeneration and OA progression (48). Conversely, the latter promotes chondrocyte apoptosis and inhibits autophagy, consequently accelerating OA progression (49). In summary, the same miRNA may target different genes, which in turn have different effects on the progression of OA.

Notably, certain miRNAs exhibit consistent expression alteration patterns in both human OA and small animal OA models. For example, protective miRNAs such as miR-140-5p, miR-214-3p, and miR-548d-5p are downregulated in surgically induced OA joints of rodents (37, 38, 41). Conversely, disease-promoting miR-128a is upregulated in both human OA and rodent OA models, and its functional intervention can alter OA progression in animals (45). This suggests that the pathological roles of these miRNAs are conserved across species. Beyond rodent models, large animal OA models have gained increasing attention in miRNA research in recent years. Large animal joints more closely resemble human joints in terms of size, weight-bearing capacity, cartilage thickness, and natural disease progression, enabling better simulation of the physiological effects of miRNAs within the complex joint microenvironment (50). For example, in equine OA models, multiple studies have reported abnormal expression of miR-223, miR-199a-3p, miR-27b, and other related miRNAs in synovial fluid or extracellular vesicles, closely associated with inflammatory responses, cartilage matrix degradation, and synovial fibrosis (51–53). miR-146a is upregulated in the synovial tissue of dogs with spontaneous OA and is likewise upregulated in early-stage human OA (54, 55), indicating a shared upregulation pattern that provides a valuable foundation for subsequent clinical translation. However, the trends of miRNA changes in large animals do not always align with those in humans. For instance, miR-146a is downregulated in late-stage human OA (54). Furthermore, taking miR-140-5p as an example: it is considered a protective miRNA in human OA with decreased expression in synovial fluid (40). In contrast, in equine OA models, researchers observed a significant increase in miR-140-5p within synovial fluid, accompanied by a decrease in plasma levels, and this phenomenon was interpreted as a compensatory protective response at the joint site against cartilage degradation (56).

## 4 Delivery of miRNAs for osteoarthritis therapy

**Figure 2:**
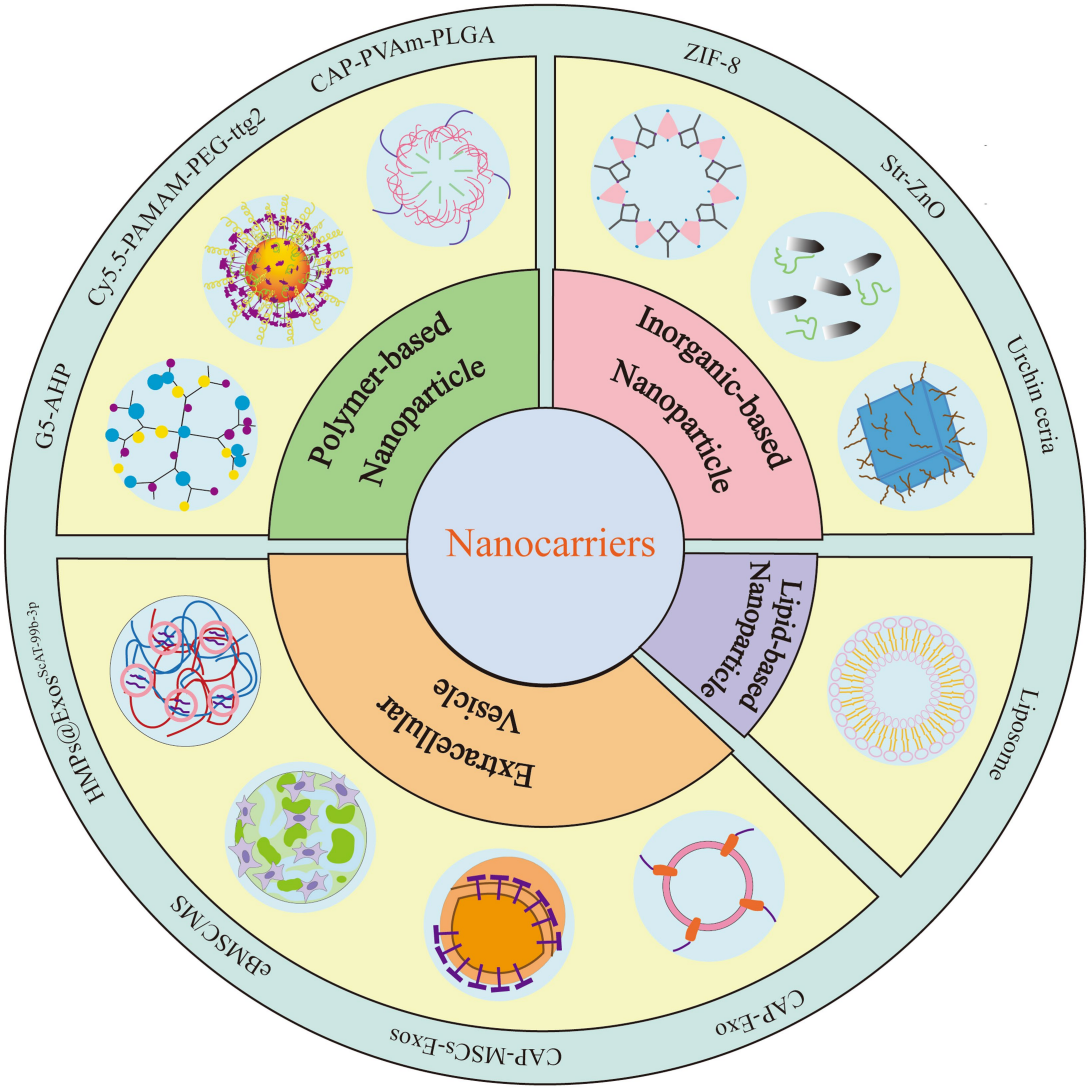
Nanocarriers for miRNA Delivery. miRNA nanocarriers for treating OA are primarily categorized into four types. The first type consists of polymer-based nanoparticles. The second type comprises extracellular vesicle delivery systems. The third type involves lipid-based nanoparticles. The fourth type utilizes inorganic nanoparticles.

The above findings indicate that miRNAs exert complex effects on the pathogenesis and progression of OA by regulating numerous biological processes, including cartilage degradation, inflammatory responses, cellular differentiation, and tissue homeostasis. These findings provide a robust foundation for employing specific modulation of their expression as a therapeutic approach for OA. In recent years, various nanomaterials have demonstrated potential applications as miRNA carriers in the treatment of OA (Figure 2; Tables 2–4).

**Table 2:** Advantages and disadvantages of various nanocarriers and findings.

Nanoparticle type	Main advantages	Main disadvantages	Findings	References
Extracellular vesicles	Excellent biocompatibility, low immunogenicity, and strong tissue penetration	Limited loading capacity and challenges in standardizing mass production	Engineered exosomes significantly enhance the targeting of miRNAs to cartilage and delivery efficiency through surface modifications such as CAP peptides. In vitro and in vivo experiments demonstrate that exosomes loaded with miR-140, miR-199a-3p, miR-223, and others effectively suppress inflammatory responses, mitigate cartilage degeneration, and significantly delay the progression of osteoarthritis	(60–64, 66, 67, 94)
Polymer-based	The preparation is relatively straightforward and has a high loading capacity	Cationic polymers may disrupt cell membranes and induce cytotoxicity or inflammation, and certain polymer degradation products may cause immune responses	Nanocarriers such as PAMAM dendrimers exhibit excellent miRNA loading capacity and controlled release capabilities. Functional modifications like PEGylation further enhance their targeting efficiency and biocompatibility. Experimental results indicate that delivering miR-224-5p, miR-445-3p, and miR-140 effectively suppresses inflammatory factor expression, reduces matrix degradation, and delays the progression of osteoarthritis	(73, 74, 77–79, 82, 83, 95, 96)
Lipid-based	The preparation process is stable, readily scalable for mass production, and highly efficient in delivery	Certain LNP components may induce inflammation and exhibit poor storage stability	Cationic liposomes and composite lipid systems can effectively encapsulate miRNAs, enhancing their stability and cellular uptake rates. The co-delivery strategy of drugs and miRNAs demonstrates synergistic anti-inflammatory and cartilage repair effects, significantly alleviating joint swelling and cartilage damage in animal models	(84, 87, 88, 97)
Inorganic-based	High surface area to volume ratio, excellent stability, and precise controllability of size and morphology	Potential cytotoxicity and poor biodegradability	Inorganic carriers such as ZIF-8 and ZnO enable efficient miRNA loading and controlled release due to their high specific surface area and structural stability. Studies indicate that inorganic carriers loaded with miR-200c-3p, miR-17-5p, and others effectively suppress inflammation, promote collagen synthesis, enhance antioxidant capacity, and demonstrate potential in recruiting stem cells and promoting cartilage regeneration	(89, 90, 92, 93, 97)

**Table 3:** Nanocarriers for miRNA delivery.

Nanoparticle type	Composition	MiRNA element	Size * [nm]	Loading/transfection efficiency	References
Polymer-based	G5-AHP	miR-224-5p	≈175	–	(77)
	CANC@miR-455-3p	miR-455-3p	≈38.2 ± 1.6	–	(78)
	ttg2-PEG-PAMAM	141/200c cluster	≈38.2 ± 1.6	–	(79)
	CAP-PVAm-PLGA	miR-140	96.4 ± 15.8	78.3 ± 9.2%	(82)
	ChoS (HA/CS)	miR-149-5p	NP^#^1 412 ± 21.18 NP2 259.45 ± 13.89 NP3 180.20 ± 10.23	NP1 84.13% NP2 73.87% NP3 60.97%	(83)
Extracellular vesicles	CAP-Exos	miR-140	40–200	≈60%	(61)
	CAP-MSCs^SC^-Exos	miR-199a-3p	90–170	≈40%	(62)
	CTP/Mir-EVs	miR-223	142.9	≈60%	(63)
	BMSC^motif + miR874^/MS	miR-874-3p	60–150	–	(64)
	CAP-EXOs/miR-148a@GAM	miR-148a	268.6	–	(66)
	HMPs@Exos^ScAT-99b-3p^	miR-99b-3p	–	–	(67)
Lipid-based	Lipo-AgPEI-miR-200c-3p	miR-200c-3p	≈130	–	(87)
	Lnxc-CL	miR-140	286.6 ± 7.3	≈100%	(88)
Inorganic-based	ZIF-8	miR-200c-3p	≈121	90.24%	(90)
	Str-ZnO	miR-17-5p	width≈30 length≈150	–	(92)
	Urchin-like ceria	miR-224-5p	≈251.8	–	(93)

*Size including miRNA. ^#^Based on varying chondroitin sulfate content, it is classified into three types of NP.

**Table 4:** Model types and induction approaches.

Composition	*In vivo* OA models	*In vitro* OA models	*In vivo* OA induction method	*In vitro* OA induction method	References
G5-AHP	Mice	Primary chondrocytes and fibroblast-like synoviocytes (FLS)	TNF-α	Destabilization of the medial meniscus (DMM) surgery and high-intensity treadmill training	(77)
CANC@miR-455-3p	Mice	Primary human OA cartilage and synovial tissues	–	DMM surgery; miR-455-3p Knockout (KO)	(78)
ttg2-PEG-PAMAM	Mice	Primary human OA chondrocytes	–	DMM surgery; miR-141/200c cKO	(79)
CAP-PVAm-PLGA	Mice	Primary mouse chondrocytes	IL-1β	DMM surgery	(82)
ChoS (HA/CS)	–	–	–	–	(83)
CAP-Exos	Rats	Primary human chondrocytes	IL-1β	DMM surgery	(61)
CAP-MSCs^SC^-Exos	Mice; rats	Primary rat chondrocytes and synovial cells	IL-1β	DMM surgery; anterior cruciate ligament transection (ACLT) surgery	(62)
CTP/Mir-EVs	Rats	Primary rat chondrocytes	IL-1β	Monosodium iodoacetate (MIA)	(63)
BMSC^motif + miR874^/MS	Rats	Primary rat chondrocytes	IL-1β	DMM surgery	(64)
CAP-EXOs/miR-148a@GAM	Mice	Primary mouse chondrocytes, human OA cartilage explants	IL-1β	ACLT surgery	(66)
HMPs@Exos^ScAT-99b-3p^	Mice	Primary mouse chondrocytes	IL-1β	DMM surgery	(67)
Lipo-AgPEI-miR-200c-3p	–	ATDC5	LPS	–	(87)
Lnxc-CL	Rats	–	–	Papain	(88)
ZIF-8	–	CHON-001	LPS	–	(90)
Str-ZnO	Rats	Primary rat chondrocytes, rat bone marrow mesenchymal stem cells	IL-1β	cylindrical cartilage defect	(92)
Urchin-like ceria	mice	Primary mouse chondrocyte	TNF-α	DMM surgery and high-intensity treadmill training	(93)

### 4.1 Extracellular vesicle delivery systems

Extracellular vesicles (EVs) are cell-derived nanoparticles encapsulated by a lipid bilayer membrane that can encapsulate various bioactive substances, such as nucleic acids, proteins, and lipids (57). Exosomes are a subtype of EVs derived from the endosomal pathway that play a crucial role in intercellular communication and signal transduction (58, 59). EVs offer considerable potential for targeted delivery of miRNA therapies for OA owing to their inherent biocompatibility, low immunogenicity, and cytotoxicity (60). Liang et al. (61) fused cartilage-targeted peptides (CAPs) with lysosome-associated membrane glycoprotein 2b on the surface of exosomes, achieving efficient encapsulation and specific delivery of miR-140, effectively alleviating cartilage degeneration and disease progression. Additional researchers have utilized CAP-modified exosomes derived from subcutaneous fat-derived mesenchymal stem cells (MSCs^SC^-Exos) to deliver miR-199a-3p, significantly enhancing the effect of cartilage repair (62). Furthermore, Liu et al. (63) constructed a dual-engineered EVs by loading exogenous miR-223 into human umbilical cord mesenchymal stem cell-derived EVs (hUC-EVs) using electroporation and combining it with surface-modified collagen II-targeted peptides, which significantly enhanced its cartilage targeting ability and therapeutic efficacy. Recently, Wu et al. (64) constructed an engineered stem cell cluster to continuously deliver miRNA-874-3p using EVs secreted by bone marrow-derived mesenchymal stem cells to achieve chondrocyte repair and regeneration while effectively mitigating the progression of OA.

Microfluidic technology has shown great potential in the field of drug delivery because of its ability to achieve precise control of droplet volume, morphology, and internal structure (65). Yang et al. (66) prepared degradable gelatin microspheres (CAP-EXOs/miR-148a@GAM) loaded with CAP-modified exosomes on the basis of microfluidic technology for the continuous delivery of miR-148a for the treatment of OA, significantly promoting the synthesis of ECM and delaying the progression of OA by inhibiting ROBO2 expression and the MAPK signaling pathway. Hydrogel microparticles (HMPs) based on hyaluronic acid prepared by microfluidic technology encapsulate extracellular vesicles derived from subcutaneous adipose stem cells loaded with miR-99b-3p to form the HMPs@Exos^ScAT-99b-3p^ system; research by Yin et al. (67) reported that this system not only addresses the issues of a short *in vivo* half-life and poor targeting of EVs but also promotes cartilage self-repair and homeostasis restoration by specifically inhibiting ADAMTS4, thereby demonstrating significant therapeutic potential for OA both *in vivo* and *in vitro*.

In the field of biological therapy research for OA, large animal studies based on secretome analysis are gradually emerging. Presently, multiple studies in dogs and horses have employed intra-articular administration of MSC secretome or blood cell secretome (BCS) to reduce inflammatory responses, alleviate joint swelling, and improve joint function (68–70). Other studies have shown that injecting human MSC-derived exosomes into porcine knee weight-bearing osteochondral defect models can significantly improve the morphological and mechanical repair of cartilage and subchondral bone, indicating that they exert cross-species effects (71). As a key component of the secretome, EVs contain abundant endogenous miRNAs (72), which may constitute one mechanism underpinning their therapeutic effects. These studies provide robust translational evidence for future engineered EV delivery systems loaded with specific miRNAs.

### 4.2 Polymer-based nanoparticles

In comparison with EV delivery systems, polymeric nanoparticles (PNPs) demonstrate highly controllable physicochemical properties, along with higher production efficiency and enhanced drug loading capacity (73, 74). Polyamidoamine (PAMAM) dendrimers are extensively employed in drug delivery because of their highly branched architecture and internal cavities (75), and polyethylene glycol (PEG) modification can optimize their surface charge and stability (76). Chen et al. (77) delivered miR-224-5p using fifth-generation PAMAM (G5-PAMAM) functionalized with arginine, phenylalanine, and histidine, thereby alleviating cartilage degeneration and synovial inflammation by inhibiting pentraxin 3. Long et al. (78) utilized 50% PEG-modified G5-PAMAM dendrimer macromolecules loaded with miR-455-3p and combined with CAPs and minimal self-peptides (MSPs), which were then further complexed with a thermosensitive hydrogel. This composite not only achieved sustained release and precisely targeted delivery of miR-455-3p but also suppressed chondrocyte hypertrophy and apoptosis while promoting matrix synthesis by regulating key pathogenic pathways. Ji et al. (79) employed PEGylated PAMAM dendritic carriers modified with chondrocyte-specific nucleic acid aptamers (tgg2) to deliver miR-141/200c cluster inhibitors. This approach relieved the suppression of SIRT1, enabling SIRT1 to inhibit the IL-6/STAT3 pathway. Consequently, inflammation was suppressed, the ECM and cartilage were protected, and cartilage degeneration was even reversed, thereby effectively delaying the progression of OA.

Polyvinylamine (PVAm), as a hydrophilic cationic polymer, can effectively bind negatively charged RNA and exhibits good transfection efficiency, making it highly promising for use in the field of targeted therapy (80). Poly(lactic-co-glycolic acid) (PLGA) is a hydrophobic polymer that has been extensively studied because of its excellent biocompatibility, biodegradability, and good mechanical properties (81). Zhao et al. (82) achieved chondro-targeted delivery of miR-140 via CAP-PVAm-PLGA, a copolymer formed from CAP-modified PVAm and PLGA, thereby suppressing inflammation and matrix degradation. Another study employed triple polysaccharide nanoparticles composed of chondroitin sulfate, chitosan, and hyaluronic acid to deliver miR-149-5p. This approach not only effectively transfected cells and significantly reduced the expression of the target gene FUT-1, but also promoted cartilage differentiation through the components released upon degradation (83).

### 4.3 Lipid-based nanoparticles

Lipid nanoparticles (LNPs) possess excellent biocompatibility and ability to improve drug stability and targeting efficiency (84), and they are widely used for delivering nucleic acids and small-molecule drugs (85). The presence of positively charged hydrophilic head groups in cationic lipids promotes electrostatic interactions with negatively charged nucleic acids, thereby enhancing the encapsulation of nucleic acids and promoting intracellular escape (86). Zheng et al. (87) encapsulated AgPEI nanoparticles loaded with miR-200c-3p within liposomes to form the Lipo-AgPEI-miR-200c-3p complex, which effectively blocked the inflammatory-apoptotic-matrix degradation malignant cycle of chondrocytes *in vitro*. However, its *in vivo* efficacy and safety require further verification. Additionally, He et al. (88) utilized lornoxicam cationic liposomes to load miR-140, obtaining cationic liposomes coloaded with lornoxicam and miR-140, which combine anti-inflammatory and gene therapy functions. This significantly improved the stability of miR-140 and its uptake efficiency by chondrocytes, effectively alleviating joint swelling, inhibiting inflammation, and promoting cartilage repair. This finding encourages the combination of other drugs with miRNA delivery for synergistic treatment of OA.

### 4.4 Inorganic-based nanoparticles

Compared to organic nanoparticles, inorganic nanocarriers exhibit superior structural stability and enhanced mechanical properties, thereby attracting significant attention from researchers (89). Yang et al. (90) utilized ZIF-8, a metal–organic framework material featuring noncytotoxic zinc(II) as its metal coordination center, as a miRNA delivery vehicle. Through a Y-shaped microfluidic chip, the researchers efficiently prepared miR-200c-3p@ZIF-8, significantly reducing the expression of inflammatory factors in OA. However, traditional spherical nanoparticles have limited loading capacity and insufficient transfection efficiency (91); thus, researchers have developed new inorganic nanocarriers. For example, Li et al. (92) delivered miR-17-5p using streamlined ZnO nanoparticles combined with methyl acrylate-modified gelatin (GelMA). In this way, bone marrow mesenchymal stem cells are recruited via zinc ions, promoting matrix synthesis while inhibiting catabolic processes. Chen et al. (93) used urchin-like cerium dioxide nanoparticles to efficiently load miR-224-5p, and their surface area increased, which not only improved their miRNA loading capacity and antioxidant capacity but also enhanced their therapeutic efficacy. Taken together, these findings indicate that the novel nanoparticle-based miRNA carriers demonstrate superior loading capacity and can effectively enhance the therapeutic efficacy of OA treatment. However, the long-term biological safety of such materials requires further investigation.

### 4.5 Comparative analysis and limitations

EVs exhibit exceptional biocompatibility, low immunogenicity, and excellent tissue penetration. However, their large-scale production remains constrained by technical challenges, with complex purification processes that are difficult to standardize (60). Furthermore, EVs possess limited cargo capacity, and the potential risks associated with engineering modifications remain uncertain. EVs derived from different cell sources may carry bioactive molecules, potentially triggering uncontrollable biological effects (94). In comparison, PNPs are relatively simple to prepare, less costly, and exhibit higher loading efficiencies (73). However, many cationic polymers carry positive charges that may cause cell membrane damage and cytotoxicity, potentially triggering inflammatory responses (95). Additionally, degradation products from certain polymers may also induce cytotoxicity and immune reactions (96). LNPs represent one of the most mature nucleic acid delivery platforms currently available and have been extensively utilized in mRNA vaccines (85). LNPs offer advantages such as stable preparation processes, ease of large-scale production, and high delivery efficiency (84). However, they still carry potential toxicity risks. In addition, due to the susceptibility of lipid membranes to oxidation and hydrolysis, they typically require cryopreservation or cold storage to ensure stability (97). Inorganic nanoparticles possess unique physicochemical properties such as large surface area, excellent stability, and precise controllability of morphology. Their primary concern lies in insufficient long-term biosafety because some inorganic nanoparticles degrade slowly or not at all within the body, potentially accumulating in tissues and triggering toxicity or inflammation (98).

## 5 Challenges in clinical translation

Although miRNA delivery based on nanocarriers shows great promise in the treatment of OA, its clinical translation and application still face multiple challenges. First, OA is a chronic condition requiring long-term, repeated administration of medication. While most nanomaterials demonstrate relative safety in short-term animal studies, their prolonged use may exacerbate local inflammation or cause chronic synovial irritation. Degradation products from the carrier may also accumulate within the joint cavity, posing potential toxicity to chondrocytes (99). Second, the multi-target nature of miRNAs themselves, coupled with the barrier effect posed by the dense articular matrix, results in coexisting off-target risks and delivery efficiency challenges (19, 100). Although previous studies have employed targeted peptides or surface modifications to enhance tissue affinity, achieving sustained and specific delivery remains challenging in the dynamically updated joint microenvironment (101). Third, challenges in large-scale production and quality control. Issues such as batch-to-batch variability in vesicles and insufficient stability of polymers and liposomes severely limit the feasibility of clinical translation (61, 102). Finally, the limitations of drug efficacy evaluation systems also constrain translational prospects. Clinical approval requires substantial, reliable preclinical and clinical data. However, most existing studies rely on rodent models, which differ markedly from humans in joint size, biomechanical properties, and disease progression (103). Future efforts should strengthen large animal studies, particularly spontaneous large animal OA models, to more accurately assess long-term drug safety, pharmacokinetic characteristics, and functional improvements. Therefore, challenges ranging from safety and biological complexity to production processes and model evaluation collectively constitute critical hurdles that must be overcome before nanoparticle-based miRNA delivery therapies can advance to clinical application.

## 6 Future perspectives

Nanocarrier-based miRNA delivery strategies demonstrate significant potential in the treatment of OA, yet several critical research gaps remain to be addressed. Future studies should delve into material safety, disease heterogeneity, and clinical translational feasibility, striving to develop novel smart delivery systems. First, biosafety assessment remains one of the most significant research gaps. Future efforts should establish a long-term, comprehensive safety evaluation system and develop novel materials with enhanced biodegradability and biocompatibility to mitigate immunogenicity and tissue accumulation risks. Second, future studies should aim to develop various hybrid nanoparticles and enhance the fine-tuned design of their functions, integrating the advantages of different materials to ultimately achieve composite nanomaterials with high targeting capability, excellent manufacturability, and superior safety. Third, developing smart nanocarriers with active response capabilities will transcend traditional passive targeting mechanisms. By incorporating smart stimulus-responsive elements that dynamically respond to changes in the OA microenvironment, researchers can achieve controlled release of miRNAs based on the unique pathological cues of OA joints. Fourth, given the multifactorial nature of OA, the therapeutic potential of combination therapies should be explored. Co-delivery of multiple miRNAs or the combined delivery of miRNAs with small-molecule drugs can synergistically modulate gene networks while simultaneously addressing multiple pathological pathways. Finally, by integrating cutting-edge technologies such as multi-omics analysis and single-cell sequencing, researchers can precisely decipher the regulatory network of miRNAs in the pathological progression of OA. On the basis of the miRNA expression profile, future studies could further leverage advanced nanotechnology platforms to achieve true personalized precision treatment.

## 7 Conclusion

In conclusion, the targeted delivery of nanotechnology-based miRNAs opens new avenues for treating OA. Overcoming delivery barriers and an in-depth understanding of disease mechanisms hold promise for developing next-generation personalized precision treatment regimens for OA patients.
